# Advances in Powder-Filled Mold Processes: A Comprehensive Review and Outlook

**DOI:** 10.3390/ma17225476

**Published:** 2024-11-09

**Authors:** Pengyu Bai, Shuhua Yang, Yalin Yan, Dongliang Wang, Yanwei Ma

**Affiliations:** 1 School of Material Science and Engineering, University of Jinan, Jinan 250022, China; 2 Institute of Electrical Engineering and Advanced Electromagnetic Drive Technology, Qilu Zhongke, Jinan 250013, China; 3 Key Laboratory of Applied Superconductivity, Institute of Electrical Engineering, Chinese Academy of Sciences, Beijing 100190, China; 4 University of Chinese Academy of Sciences, Beijing 100049, China

**Keywords:** die filling, powder flow, uniform fill, powder-filling process

## Abstract

Powder molding technology is a versatile process widely used in the pharmaceutical, ceramic, chemical, food, and powder metallurgy industries. The powder-filling mold process is a key link in powder compression molding, and the uniformity and consistency of powder filling directly affect the final quality of powder products. Powder filling of molds is a more complex flow process. This paper first reviews the methods used to test powder flow characteristics and comments on their applicability to the mold-filling process, provides an in-depth discussion of four different filling techniques, focusing on the flow behavior of the powder during the filling process, and analyzes the effects of powder characteristics and process parameters on the filling effect. By reviewing the latest advances and identifying the key challenges, a valuable reference is provided for the mold-filling process.

## 1. Introduction

Powder-pressing molding technology is a universal powder processing technique widely applied across various industries, such as pharmaceuticals [1,2,3,4], ceramics [5,6,7,8], chemicals [9,10,11], food [12,13,14], and powder metallurgy [15,16]. As global manufacturing innovation and processing scale continue to expand, the application of powder molding technology across industries is becoming increasingly widespread. The demand for product performance is also rising, with conventional methods of producing powder products often resulting in defects such as voids [17,18,19], cracks [20,21], and uneven textures [22]. Therefore, meeting the requirements for uniformity, densification, high precision, and strong shape adaptability has become the direction of development for powder molding technology. The powder pressing process generally includes three stages [23]: mold filling, pressing, and ejection, where the powder mixture is filled into the mold cavity either through gravity or other external forces, compressed under high pressure into a continuous dense structure, and finally ejected from the mold. These three stages have a significant impact on the final performance of the powder compact [24], especially the process of powder filling into the mold cavity.

In 1998, Demetry et al. used tactile sensors to measure the density distribution of powder filling in the mold before and after pressing, discovering that the post-pressing density distribution was consistent with that before pressing, indicating that the pressing process could not eliminate the adverse factors during mold filling [25]. In 2008, Korachkin et al. studied the impact of unevenness during the filling process on the results of pressing, showing that unevenness during the mold-filling process significantly increased variation in pressing stress and density gradient [26]. Therefore, the powder-filling process (mold filling) is key to powder-pressing molding. The densification and uniformity of powder filling directly affect the subsequent pressing process and, consequently, the final quality and performance of the powder products. The powder loading process involves filling a certain amount of powder into the mold, ensuring that the filled powder is densely and reasonably distributed within the mold cavity uniformly. The majority of actual powder production processes adopt a free-filling method [27,28,29,30,31], where a certain amount of powder is poured into the mold, and the powder loosely fills the cavity solely under the action of gravity coupled with the effect of the geometric properties of the powder; there are often a large number of voids between adjacent powders, with the loose packing density being only 30–65% of the theoretical density. The size and shape of the voids affect the density and hardness of the green body, leading to defects such as cracks and dimensional deformation during subsequent processing. The presence of defects affects the strength [32], hardness [33], toughness [34], ductility [35], dimensional stability [36], and other mechanical properties of the finished product. The complexity of powder mold filling stems from the interaction of multiple variables, including the physical properties of the powder [37,38], environmental conditions [39,40], filling methods [41,42], etc. In addition, the design of the mold is critical, and the shape [37], size [43], and surface quality of the mold [44] can significantly affect the flow of powder and filling efficiency. Accurate mold design can reduce friction in the powder-filling process to improve mobility and achieve uniform filling. Therefore, revealing the densification and uniformity mechanism of powder filling, understanding the flow process of mold-filling powder, exploring the impact of powder characteristic parameters and powder-filling process parameters on powder filling, and providing reference data for improving and optimizing the powder-filling process is of crucial importance for enhancing the performance of powder products.

In recent years, along with the development of materials science and engineering technology, the research of powder mold filling has gradually moved toward intelligence and automation, combined with computer simulation and emulation, which can help us better understand the dynamic changes in the filling process and then formulate the best filling strategy [42,45,46,47]. In addition, the continuous emergence of new materials, such as composite materials [48], high entropy alloys [49], and functional gradient materials [50], further stimulate the research needs of powder mold technology; in the face of how to ensure the performance of new materials under the premise of achieving high-efficiency filling, researchers are actively exploring new filling methods and technical means. Although there has been much research focusing on various aspects of powder mold filling, there is relatively little literature that systematically integrates and summarizes the results of this research. In order to fill this gap, this paper aims to provide a comprehensive review of the related theories and technologies of powder mold filling, discuss its influencing factors in detail, analyze the key problems and challenges in the field at present, and look forward to the direction of future development in order to provide theoretical guidance and practical references on the filling of powder molds to support the development of high-performance materials.

In this paper, the research background of the powder-filling process is briefly introduced in Section 1; Section 2 outlines the flow characteristics of powder during the mold-filling process and reviews the assistance of different flowability testing methods in the mold-filling process; Section 3 focuses on the flow behavior of powder under different filling methods; and, finally, Section 4 concludes with an outlook on the powder-filling process.

## 2. Powder Flowability in Mold-Filling Process

During the powder-filling process, powder flowability is an important evaluation parameter in industrial production [51,52,53,54,55,56,57]. To achieve consistent and uniform filling outcomes, freely flowing powders are preferred, as poor flowability can lead to product uniformity issues and unnecessary economic losses in industrial production. Therefore, understanding the flow characteristics of powder is crucial.

Powder flowability is not an inherent property of the powder particles but a derivative characteristic closely related to the powder’s physical properties and flow environment parameters, such as powder particle size [58,59], size distribution [60], shape [58], moisture content [61,62], and interparticle forces [63,64]. Generally, powders with larger particle sizes and narrower size distributions exhibit better flow properties, displaying lower interaction behaviors when larger particles contact each other. As particle size decreases, van der Waals forces begin to overcome gravity, leading to higher adhesion and cohesion between adjacent particles and resulting in particle agglomeration. Typically, spherical particles have smaller contact areas, smoother morphologies, and are more prone to sliding past each other, thus exhibiting better flowability. The impact of moisture on powder flow characteristics depends on the moisture content; on the one hand, when the moisture content is low, the water molecules between particles can reduce interparticle friction, acting as a lubricant and eliminating static electricity and positively affecting powder flow. On the other hand, as moisture content increases, the water adsorbed on the particle surfaces forms liquid bridges and generates capillary forces, causing the powder fill volume to increase and small particles to agglomerate into irregularly shaped larger particles, worsening powder flowability. The aforementioned parameters ultimately affect interparticle forces, such as electrostatic forces, capillary forces, van der Waals forces, cohesion, and friction, which interact with each other and ultimately determine the powder’s flow characteristics [65].

**Figure 1:**
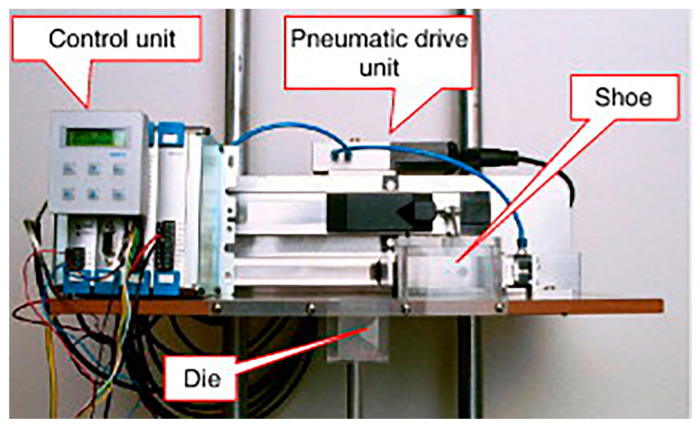
A model die-filling system [67].

As can be seen in the above discussion, assessing powder flowability is a complex issue, and a single measurement parameter cannot accurately evaluate powder flow properties. Although various methods for evaluating powder flowability exist in the industry, finding a universal characterization method suitable for all applications is challenging. Powder flowability rankings can change across different evaluation systems, and linking test results to actual powder flow behavior is very challenging. The mold-filling process is an instantaneous process, and the flow behavior of powder is quite complex. To better study the scientific issues during the mold-filling process, Wu et al. developed a model system consisting of a fixed mold and a moving feeding mechanism to simulate the powder-filling process. This system comprises transparent molds and boots, using a high-speed video system to observe and record the filling process, as shown in Figure 1 [37]. In this system, the filling ratio of the cavity is defined as:

(1) $$\delta =\frac{m_{x}}{M},$$

where *m_x_* is the mass of powder filled into the cavity and *M* is the mass of powder when the cavity is fully filled. The filling ratio is used to evaluate mold-filling efficiency, and the concept of “critical filling speed” is introduced. The relationship between the filling ratio and the charging boot’s movement speed is given by the formula:

(2) $$\delta ={\left(\frac{\nu_{c}}{\nu_{s}}\right)}^{n}\;,$$

where *v_c_* is the critical movement speed of the charging boot, *v_s_* is the actual movement speed of the charging boot, and *n* is the exponent [66]. The critical filling speed is used to evaluate powder flow performance, indicating the maximum filling speed that allows the powder particles to fully fill the cavity. When the filling speed exceeds the critical speed, the powder cannot completely fill the cavity; when the filling speed is less than the critical speed, the cavity is fully filled (i.e., the higher the critical filling speed, the better the flowability of the powder particles [37]).

Over the past few decades, various innovative methods for evaluating powder flow have been developed. Therefore, understanding the different evaluation methods for powder flowability and their interrelationships, as well as making reasonable use of powder flow characterization techniques, is crucial. Table 1 outlines different evaluation methods and their scopes of application, reviewing their assistance in the powder-filling process. Despite the existence of multiple testing methods to evaluate the flowability of powder during the mold-filling process, there are still some limitations that need to be further explored and improved to provide more comprehensive flowability data.

## 3. Powder-Filling Methods

### 3.1. Gravity Filling

In the actual production process, powder filling is mostly done through gravity filling, where the powder mixture is poured into the mold from a feeder located above. The powder mixture is deposited into the mold cavity solely under the influence of gravity. This method of filling has been extensively studied over the past two decades [41,67,83,84,85].

**Figure 2:**
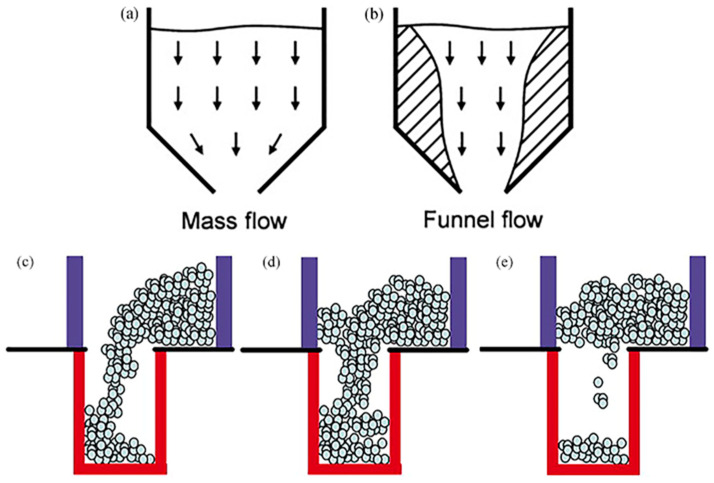
Powder flow patterns in the hopper: (**a**) mass flow, and (**b**) funnel flow [86]; and types of powder flow in gravity filling: (**c**) nose flow, (**d**) bulk flow, and (**e**) intermittent flow [39].

Gravity filling typically utilizes a hopper for feeding. Research on powder flow within hoppers is widespread. Broadly speaking, powder flow within hoppers can be divided into two modes—“mass flow” and “funnel flow”, as illustrated in Figure 2a,b [46,86]. In mass flow, all powder moves simultaneously, adhering to the “first-in, first-out” principle. In contrast, during funnel flow discharge, the powder is divided into two regions: a central flowing region and a stagnant region near the walls [37]. Powder from the central flowing region leaves the hopper first, followed by powder from the stagnant region. This occurs because the powder in the central flowing zone experiences the same pressure at the top and bottom, allowing for free fall under gravity. The powder in the stagnant region is influenced by the angle of the hopper and wall friction and exits subsequently [87,88]. Mass flow exhibits stability and lacks segregation, representing an almost ideal flow mode. On the other hand, funnel flow results in uneven powder distribution and is prone to segregation and separation [46,89,90]. Moreover, the discharge process from the hopper is not merely a singular flow mode, but a transition from mass flow to funnel flow occurs [46,91,92,93], indicating the existence of a flow transition zone.

The analysis above suggests that the flow behavior in funnel flow mode is more complex, sparking research interest. When stagnant zones exist in funnel flow, the flow process resembles that of a shoe feeder system [37]. Utilizing shoe feeder systems, numerous scholars have systematically studied the flow behavior of powder during gravity filling. Wu et al. discovered that during the movement of the shoe feeder, powder can exhibit two flow modes: nose flow and bulk flow [23,37], as shown in Figure 2c–e [39]. When the shoe feeder, carrying a fixed mass of powder, slides over the mold, powder moves toward the rear end of the shoe due to friction between the powder and the shoe bottom, and the shoe feeder’s initial acceleration, forming a nose-like profile. Powder from the upper region of the shoe feeder can deposit into the mold cavity through this profile, termed “nose flow”, as depicted in Figure 2c. In scenarios of high speed or smaller mold openings, the nose profile swiftly slides through the mold opening, allowing the powder to fall freely from the shoe feeder bottom into the mold cavity. If this flow is continuous, it is termed “bulk flow”, as shown in Figure 2d. During gravity filling, nose flow and bulk flow are the primary modes of powder movement. Nose flow predominates at lower shoe feeder speeds, while bulk flow takes precedence at higher speeds [39]. They also found that nose flow rapidly expels air from the mold cavity, achieving better filling results, whereas bulk flow, due to slower air expulsion, results in lower overall powder fill rates due to the interaction between residual air and powder particles [37]. Additionally, for certain viscous materials, powder flow tends to be discrete and random, with powder often depositing into the mold cavity in large aggregations or clusters of small particles, termed “intermittent flow”, as shown in Figure 2e [39,94].

**Figure 3:**
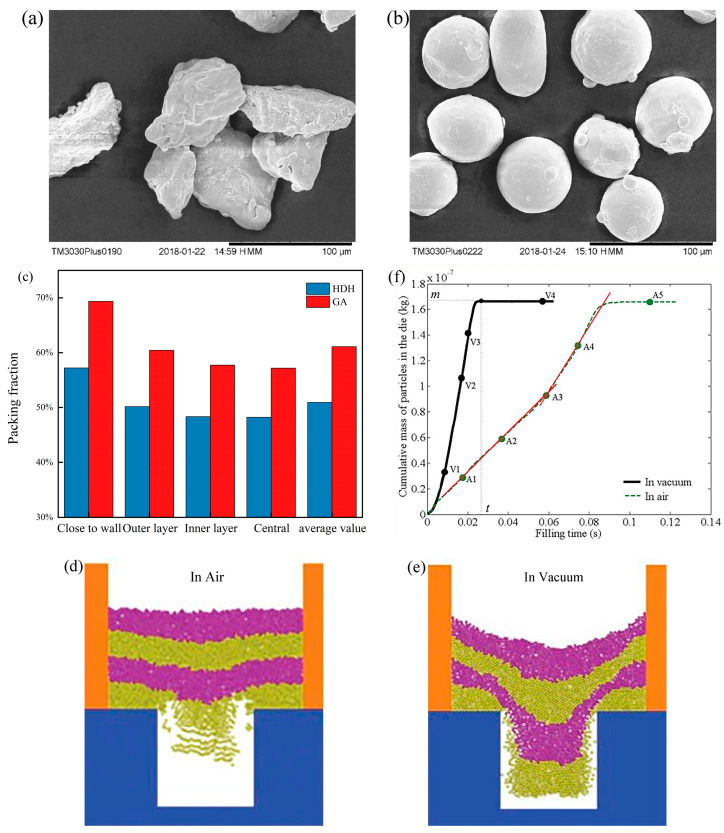
SEM images of (**a**) hydride-dehydration (HDH), and (**b**) gas atomization (GA) of Ti_6_Al_4_V samples [95]. (**c**) Filling fraction variation of HDH and GA powders along the radial region. Adapted from [95]. Powder flow patterns in air (**d**) and vacuum (**e**) at the same moment during the moldfilling process, where yellow and purple are the same particles with different colors in order to observe the macroscopic flow patterns. (**f**) Curves of the mass of particles deposited into the mold with mold-filling time during mold filling in vacuum and air [40].

Powder-filling efficiency during gravity filling largely depends on powder characteristics and process conditions. Generally, smaller particle sizes, more complex particle shapes, and greater interparticle cohesion tend to lead to arching phenomena, poorer powder flowability, and worse filling outcomes. Wu et al. found through shoe feeder systems that the fill rate decreases with decreasing particle average size and increasing irregularity of particle shape [37]. Mills et al. also confirmed this, noting that larger-particle powders have higher critical speeds and better filling outcomes under gravity filling [38]. Mehrabi et al. studied the powder fill rate of Ti_6_Al_4_V metal powders produced by two different manufacturing processes—hydrogenated dehydrogenated irregular powder (HDH) (Figure 3a) and gas atomized spherical powder (GA) (Figure 3b)—and found irregular-shaped powder (HDH) had lower fill fractions in all regions compared to spherical powder (GA) due to the tendency of irregular HDH particles to interlock, resulting in lower packing rates. However, for both samples, fill fractions increased from the central region to the walls, since the friction between powder particles and the walls was less than the interparticle friction, leading to higher packing rates near the walls and lower rates in the center (Figure 3c) [95]. Moreover, numerous studies indicate that powder flow entering the mold interacts with airflow within the mold cavity, ultimately determining powder stacking outcomes. Wu [37], Schneider [39], Guo [40,96], and others have explored the effect of airflow on mold-filling outcomes. Guo et al. compared the powder-filling outcomes of dispersed and mixed particles in air and vacuum environments in a fixed boot, using a coupled discrete element method (DEM) and computational fluid dynamics (CFD) approach, finding that air significantly impacts filling outcomes, especially for smaller or lighter powder particles. For single-dispersed particles, as shown in Figure 3d,e, powder particles exhibit better flowability in a vacuum environment at the same moment, because air tends to slow down powder flow due to adhesion forces acting on the powder particles. Figure 3f demonstrates the variation in deposited particle mass in the mold over time during filling in both vacuum and air environments. The study found that during filling in a vacuum, the filling speed remains constant for most of the time until a deceleration phase is observed, by which point the mold is almost entirely filled. In air, powder flow can be categorized into two phases: an early slow flow state and a later rapid flow state, and it is evident that filling in the air is much slower than in a vacuum [40,96]. For poly-dispersed particles, when filling in air, due to the influence of air, lighter particles fall slower than denser particles, resulting in a layer of light particles at the top of the powder layer, which, after filling, enriches in the middle of the top layer of the powder. Meanwhile, in vacuum filling, such aggregation of light particles is not observed and both types of powder particles fall at the same speed and are evenly distributed [96]. Therefore, how to more quickly expel air from the cavity during the mold-filling process becomes crucial, especially for molds with complex shapes. However, the study also found that when the particle size and density are sufficiently large, the impact of airflow can be negligible. Moreover, some scholars have proposed that airflow during the mold-filling process also has certain advantages. The airflow acts as a lubricant between powder particles, making them more likely to slide past each other, thereby improving powder flowability [97,98].

**Figure 4:**
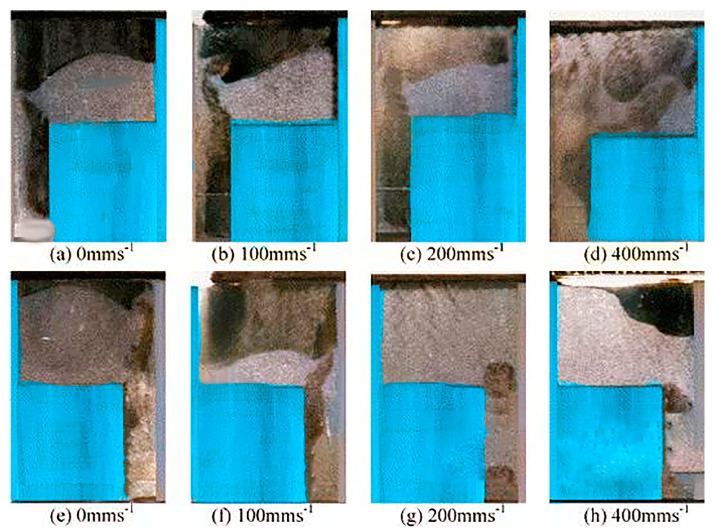
Powder flow states into a complex stepped mold at different feeding boot speeds, with the (**a**–**d**) steps on the right side and the (**e**–**h**) steps on the left side [37].

Achieving a consistent and uniform density distribution of powder in the mold-filling process is challenging. Apart from the inherent characteristics of the powder affecting the filling, the dimensions and shape of the mold also significantly impact the powder fill density, prompting many meaningful explorations. Rice and Tengzelius conducted powder-filling experiments using circular molds of different diameters and found that fill density decreased with decreasing mold diameter, and when the mold diameter was sufficiently large, the fill density remained constant [99]. Bocchini et al. studied the impact of mold width on fill density using copper powder and found that fill density increased with increasing mold width, as smaller molds are not conducive to particle rearrangement [100]. Ready et al. later also confirmed that fill density decreased as the mold aspect ratio decreased [101]. Guillot et al. found that deeper molds resulted in lower fill densities. For molds with complex shapes, the density variation is even more complicated [43]. Wu et al. used a simulated shoe system to study the effect of simple and complex molds on the powder-filling process, where the complex mold included steps of varying width. The study showed that the powder-filling process is very sensitive to the geometry of the mold cavity. When using stepped molds, the fill rate was always slow, and the fill density was lower in narrower areas. For the moving simulation shoe, the position of the steps on either side differed, further altering the powder flow pattern, as shown in Figure 4. When the narrow section is downstream of the moving shoe direction, the chimney effect of the powder is stronger, producing noticeable turbulence. When the narrow section is upstream, the chimney effect is quickly suppressed, with no significant turbulence, making the filling process gentler. Additionally, they found that when using simple molds for filling, wider molds had higher critical speeds [37] (critical shoe speed can be used as an indicator of filling efficiency; the higher the critical shoe speed, the better the flowability of the powder). However, in actual applications, product shapes are often complex and varied. Precisely designing mold shapes and dimensions to accommodate uniform powder distribution within the mold according to actual needs presents a significant challenge.

Over the past few decades, gravity-filling methods have been widely utilized across various industries due to their ease of implementation, low cost, and strong adaptability. However, ensuring the uniformity of powder within molds using gravity filling can be challenging, and the efficiency of mold filling is often low. Accordingly, the optimization of gravity-filling mold designs with respect to filling speed [41,102], filling time [41], and filling accuracy [103], with the objective of achieving higher filling density and minimizing powder segregation within the mold, represents a pivotal challenge. In this regard, utilizing computer modeling for computational analysis has shown great potential, For gravity filling of the material to be determined, the filling process was simulated by coupling the discrete element method (DEM) and computational fluid dynamics (CFD) using software such as ANSYS Fluent 2023 R1, OpenFOAM 10, and COMSOL Multiphysics 6.0 [104,105,106,107,108,109], and the effect of different process conditions on filling uniformity and product quality was evaluated by using different settings (e.g., filling speed, filling height). At the same time, actively exploring and innovating new powder-filling methods presents a promising new direction.

### 3.2. Suction Filling

**Figure 5:**
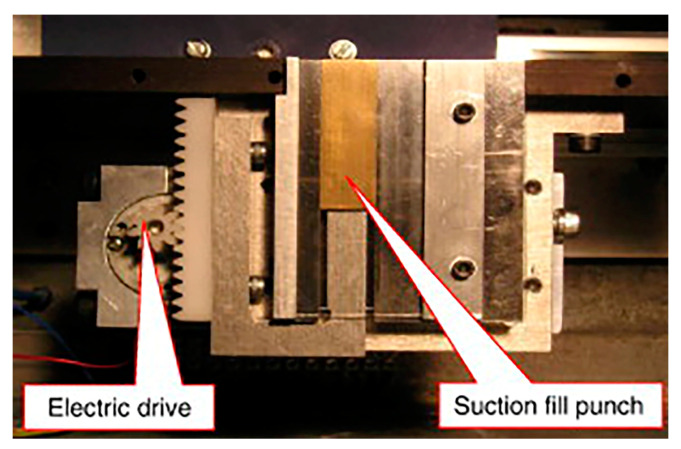
Model of the pumping filling schematic diagram of the system [67].

Due to reliance solely on gravity for powder filling into mold cavities, the filling rate is relatively low, and the impact of air on powder-filling effectiveness is significant. As more powder fills the mold, the air pressure within the cavity increases, severely hindering the efficiency of powder filling [40,96]. To improve the powder-filling outcome, Jackson et al. devised a suction-based forced powder-filling method. In this approach, a movable piston is installed within the mold cavity, and the downward movement of the piston applies a suction force on the powder to accomplish the filling process, as illustrated in Figure 5 [67]. Studies have shown that compared to gravity-based filling, suction filling can significantly increase the mold-filling rate by approximately 2.5 times [110].

**Figure 6:**
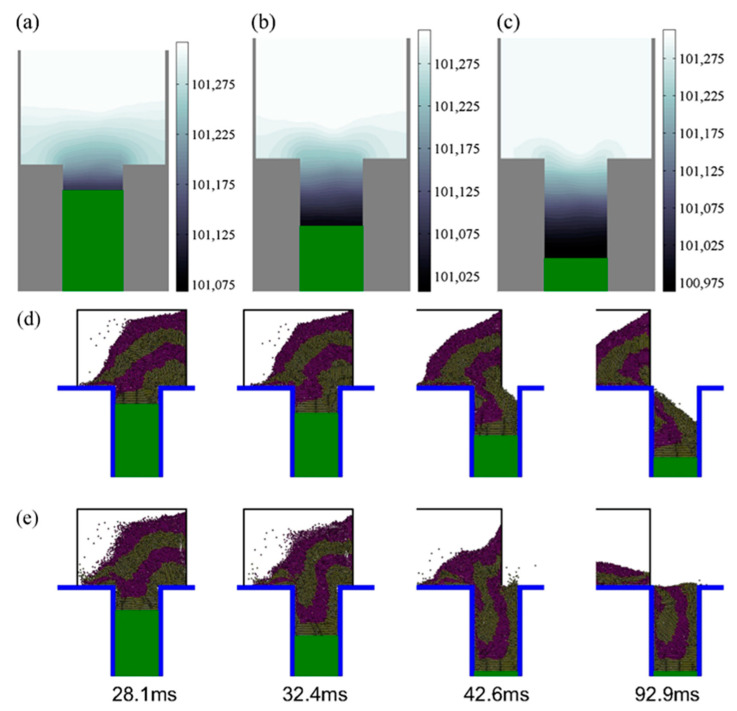
Air pressure distribution at different moments when suction filling is carried out at a piston speed of 100 mm/s: (**a**) t = 8.5 ms, (**b**) t = 21.3 ms, and (**c**) t = 31.5 ms. Powder flow state at different piston speeds when suction filling is carried out at a feed shoe speed of 140 mm/s: (**d**) piston speed of 100 mm/s, and (**e**) 276 mm/s piston speed [45].

During the suction-filling process, initially, the absence of residual air in the mold cavity does not hinder the deposition of powder into the mold. On the other hand, due to the suction effect, a significant pressure difference is created above and below the powder bed, thereby accelerating the powder-filling rate. Wu and Guo et al. simulated the flow process of powder in the suction-filling mode. The simulation results revealed that the downward movement of the piston generates a low-pressure environment beneath the powder bed (inside the mold cavity), with the pressure inside the cavity gradually decreasing from top to bottom, as shown in Figure 6a–c. This creates a pressure gradient over the powder bed, further promoting powder flow, while the pressure distribution remains relatively uniform in the horizontal direction [45].

The suction-filling process typically involves simultaneous powder feeding and suction. Therefore, for the suction-filling system, the relationship between the feeding speed and suction speed determines the powder-filling behavior. In initial experimental studies, the piston could only fall freely under the influence of gravity, uncontrollably. Subsequently, Sinka et al. improved and upgraded this mode by driving the piston with an electric device to conduct deeper studies on suction-filling conditions. When the suction speed is set to a constant 100 mm/s, whether in air or vacuum, the critical speed for suction filling is much higher than that for gravity filling, and a high filling rate is maintained when the shoe feeder speed exceeds the critical speed [67]. Wu et al. also studied the effect of different suction speeds on the filling process using a moving shoe feeder. When the shoe feeder moves at a constant speed and the suction filling is performed at a lower suction speed of 100 mm/s, the piston has not reached the bottom of the mold cavity by the time the shoe feeder completely passes the mold opening, resulting in incomplete filling of powder particles, as shown in Figure 6d. However, by increasing the suction speed to 276 mm/s, the piston can reach the bottom before the shoe feeder completely passes the mold opening, achieving complete powder filling, as shown in Figure 6e [45]. Zakhvatayeva et al. later proposed the existence of a critical ratio between the feeding speed and suction speed, i.e.,

(3) $$V_{CR}=\frac{V_{f}}{V_{s}}$$

where *V_f_* is the feeding speed, and *Vs* is the suction speed, meaning when suction filling operates at this critical speed ratio, the piston exactly reaches the bottom of the mold cavity as the shoe feeder fully passes the mold opening, resulting in complete powder filling. Thus, suction filling can be categorized into two different powder flow states: slow filling and rapid filling. When the speed ratio *V_R_* is less than *V_CR_*, it is termed “slow filling”, where the piston reaches the bottom of the mold cavity before the shoe feeder fully passes the mold opening. Conversely, when *V_R_* is greater than *V_CR_*, it is “rapid filling”, where the piston has not reached the bottom by the time the shoe feeder fully passes the mold opening. Generally, in the slow-filling state, complete powder filling can be achieved, while in the rapid-filling state, the powder-filling rate is lower, resulting in incomplete filling [57,111]. In summary, by evaluating the ratio of filling speed to suction speed and precisely controlling the suction speed and filling speed, various powder-filling behaviors can be scientifically predicted, providing strong support for industrial manufacturing.

It is noteworthy that the permeability of the powder plays a crucial role in suction filling. Mills et al. reported the effects of particle size and density under suction filling, conducting experiments with microcrystalline cellulose of different particle sizes and densities. The results showed that, compared to gravity filling, suction filling achieves a higher critical speed and filling rate, especially for powders with smaller particle sizes and lower bulk densities. This is because the principle of suction filling differs from that of gravity filling, where powder permeability plays a key role. Small and irregular powder particles generally have lower permeability, making it easier for small particles to fill the gaps between each other. During suction, the applied suction force significantly affects the powder, whereas large spherical particles typically have high permeability, allowing air to pass more easily through the gaps between particles, with less impact from the suction force [38].

**Figure 7:**
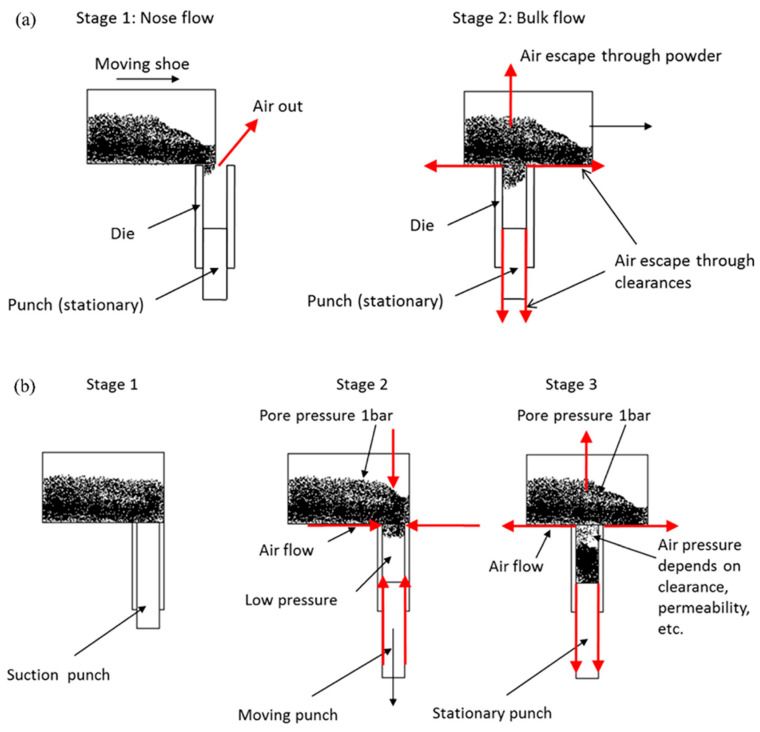
Schematic diagram of airflow mechanism during gravity filling (**a**) and suction filling (**b**) [41].

Moreover, during the suction-filling process, the airflow within the mold cavity significantly differs from that in gravity filling. As depicted in Figure 7, in gravity filling, air can only be expelled through the tiny gaps between powder particles or system gaps. As the powder fills up, the gaps between particles gradually reduce, making it difficult to expel air, and the air pressure inside the cavity gradually increases, eventually leading to incomplete powder filling or uneven density distribution. In suction filling, the downward movement of the piston creates a negative pressure state in the mold cavity, promoting powder filling. During this stage, due to the pressure effect, air enters the mold cavity through system gaps or powder gaps. However, once the piston moves to the lowest end of the mold cavity, ending the suction process, powder then fills the mold under the influence of gravity, and air can only be expelled through the gaps, leading to a corresponding increase in cavity air pressure, thereby inhibiting further powder filling. Nonetheless, due to the negative pressure effect during the initial suction process, the filling outcome is usually better than pure gravity filling [38,41]. To improve this, mold design can be optimized by adding exhaust channels or using mold materials with good ventilation, such as special metals or porous ceramics, to further enhance airflow. Additionally, a vacuum-assisted exhaust system can be used during the filling process to reduce air retention, lower air pressure, and thus improve the filling outcome.

In conclusion, the powder suction-filling process is an efficient and precise powder-filling technique. By creating a negative pressure, it enables rapid and uniform filling of the mold cavity, reducing the formation of bubbles and voids, thereby enhancing the density and uniformity of the product. This process is applicable to a variety of powder materials, and is especially suitable for powders with small particle sizes, high viscosity, and poor permeability. Through continuous technological innovation and process optimization, integrating intelligent control systems and online monitoring technologies, and real-time prediction of powder flow behavior, the suction-filling process continues to show great development potential for the future.

### 3.3. Vibration Filling

In most technical applications, there is a need for densely packed powder. However, traditional filling methods such as gravity filling often result in the phenomenon of arching between powder particles, leading to larger internal voids and lower fill densities. Additionally, the presence of particles of different shapes and sizes in the powder can cause uneven distribution and, consequently, segregation. Common methods to increase densification include vibration, tapping, and hammering [112], with vibration being a standard densification technique. During the powder-filling process, applying a certain degree of external vibration to the piled powder particles within the mold, under the combined action of gravity and external vibration, facilitates particle displacement and rearrangement. Thus, smaller particles can fill the gaps between larger particles, increasing the powder’s fill density and ensuring a uniform distribution.

**Figure 8:**
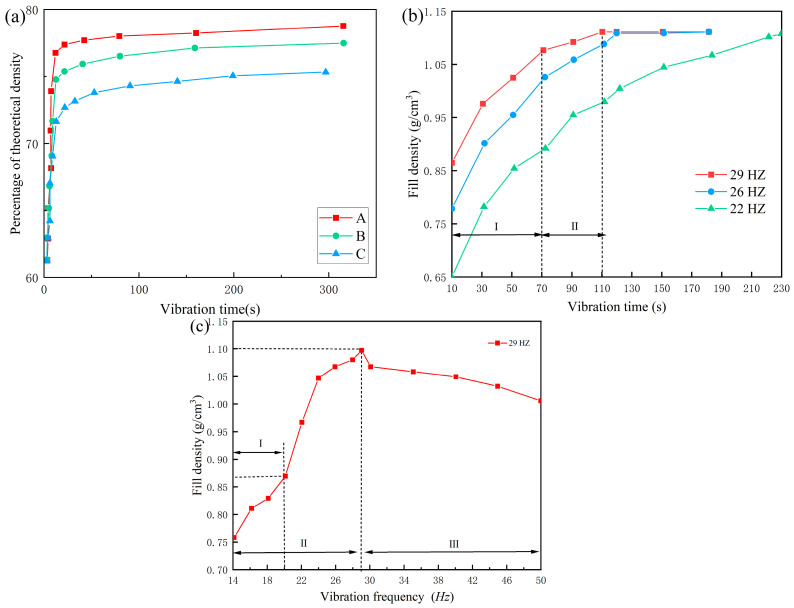
(**a**) Curves of the theoretical filling density of powder with time under different amplitudes (the amplitudes of A, B, and C in the curves are 2.6 mm, 2.0 mm, and 1.3 mm, respectively). Adapted from [113]. (**b**) Curve of filling density of Si powder with time under different vibration frequencies. (**c**) Variation curves of Si powder-filling density with vibration frequency under the same vibration time. Adapted from [115].

Optimizing vibration parameters is a critical step in enhancing the powder-filling outcome. To investigate the effect of different vibration conditions on the fill result, researchers have conducted extensive experimental studies and numerical simulations on the powder-filling process under vibration conditions. Evans et al. examined the impact of powder particle characteristics and vibration parameters on fill density during vibration filling. The study showed that applying external vibration could improve the fill density of powder, and changes in vibration parameters correspondingly altered the fill density. Figure 8a shows the theoretical fill density variation over time under the same frequency but different amplitudes, indicating that fill density increases with amplitude [113]. Knight et al. observed changes in the fill density of spherical particles within a cylindrical tube under vibration conditions and found that the powder-fill fraction increased with vibration intensity and eventually stabilized [114]. Fan et al. explored the effects of vibration time and frequency on the filling and mixing effect of Si powder, finding that powder-fill density increased with vibration time and remained constant after reaching a certain value. Furthermore, they discovered that, with an increase in vibration frequency, the time to reach a constant fill density decreased, as shown in Figure 8b, which displays the relationship between vibration time and powder-fill density at different vibration frequencies. Initially, the consolidation velocity of the powder is rapid, but it gradually slows over time, eventually ceasing, at which point the fill density stabilizes and reaches its maximum value. This is because when the powder is freely piled, interparticle arching is common, and the powder has many internal voids. In the early stages of vibration, under the combined action of gravity and external vibration, interparticle friction and collision cause displacement and rearrangement, breaking the arching effect present in loose packing and rapidly filling the internal gaps, leading to a quick consolidation velocity initially. As time progresses, the internal voids of the powder decrease, limiting particle displacement, thus gradually reducing the consolidation velocity until the interparticle gaps are essentially filled, and the fill density gradually stabilizes. Figure 8c shows the powder-fill density at different vibration frequencies over the same vibration time, indicating that, with an increase in vibration frequency, the fill density first increases and then decreases [115]. Jaggannagari et al. reported similar trends in elongated prismatic containers during particle vibration filling. At lower vibration frequencies, the interparticle forces are weaker, leading to reduced ability to fill gaps and break arching effects, resulting in lower fill density. As the vibration frequency increases, the capacity to destroy arching and fill gaps strengthens, further increasing the fill density. However, beyond a certain frequency, a noticeable “boiling” phenomenon occurs in the powder at the top of the mold cavity during vibration, causing particle stratification, and the higher the frequency, the more severe the “boiling” and stratification phenomena, leading to a decrease in fill density [116]. Hence, mid- to low-frequency vibration seems to be a good choice, providing enough energy for particle flow while avoiding the density reduction caused by excessive fluidization, thus achieving optimal filling results.

During the vibration-filling process, the particle system exhibits many fluid-like behaviors, such as the Brazil nut effect [117,118,119,120], particle convection [121,122,123], surface standing waves [124,125], arching, and separation [122,126], among others.

**Figure 9:**
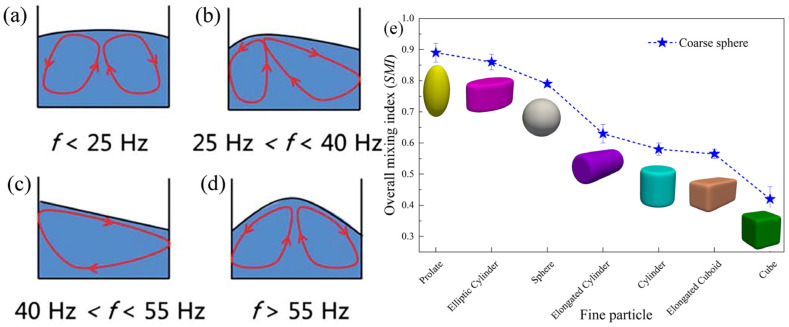
(**a**–**d**) Schematic diagrams of four convection modes under vertical vibration with different vibration frequencies [133] and (**e**) overall mixing index (SMI) of seven different shapes of particles [135].

In the transport process of Brazil nuts, after long-term vibration during transit, the larger Brazil nuts are found at the top of the storage bin, while smaller nuts have moved to the bottom, a phenomenon known as the Brazil nut effect [117,118,119,120]. Conversely, under specific vibration conditions, particle shapes, and container shapes, a reverse Brazil nut effect may occur [127]. In previous studies, both phenomena have been explained using convection [128,129] and geometric mechanisms [119,130]. In 1831, Faraday first observed convection phenomena in a vibratory particle system within a cylindrical container: particles in the center of the container moved upward, while those along the container walls moved downward [131]. Over the following decades, researchers conducted extensive and in-depth studies on particle convection, with Gallas et al. discovering different types of convection cells using molecular dynamic methods [132]. Knight et al. studied convection in cylindrical containers with glass particles, finding that the upward flow velocity along the axial direction decays exponentially from top to bottom [114]. Hu et al. reported the effect of vibration frequency on the convection modes of particle systems under vertical vibration, identifying four different convection modes as the vibration frequency varied. When the frequency f < 25 Hz, the convection mode is as shown in Figure 9a, where convection particles begin to rise from the center of the container, then disperse and move downward along the container walls, forming nearly symmetrical double convection cells. As f increases from 25 Hz to 40 Hz, a noticeable particle mound forms on the mixture surface as shown in Figure 9b, with convection particles rising but deviating from the center of the container, then dispersing and moving downward along the container walls. With further increases in frequency, the deviation of convection particles from the central axis increases. When 40 Hz < f < 55 Hz, convection particles first rise from one side of the container and then move downward on the opposite side, forming a single convection cell as shown in Figure 9c. Figure 9d displays the convection mode when f > 55 Hz, with convection particles first rising along the container’s central axis, then moving downward along the container walls, forming double convection cells. However, this mode differs from Figure 9a, featuring a protrusion in the center of the powder particle top (whereas the top in Figure 9a is relatively flat [133]). Meanwhile, Hu et al. found that vibration acceleration had little effect on the system’s convection mode. Zhang et al. also confirmed this conclusion, discussing the convection modes of particles under various vibration parameters, suggesting that vibration frequency has a significant influence on convection modes and directions, while vibration acceleration mainly affects the size and strength of convection [134]. Therefore, convection modes can be controlled by adjusting vibration frequency. Particle convection behavior has both advantages and disadvantages during the vibration-filling process. On one hand, convection behavior can promote uniform mixing of different particles, reduce void rates, accelerate the filling process, and improve filling efficiency. On the other hand, convection phenomena may lead to uneven local density distribution and accelerate equipment wear. Thus, whether convection behavior is beneficial should be evaluated based on actual application scenarios. Generally, applications that benefit from high-density filling and are insensitive to particle breakage, such as powder metallurgy and building materials, may find convection behavior advantageous. Applications that require high uniformity and particle integrity, such as pharmaceutical powders and food processing, may find convection behavior detrimental, and production adjustments in vibration frequency and amplitude can mitigate the negative impacts of convection.

In industrial applications, most particles are not standard spheres, and results from spherical particle systems studies may mislead non-spherical particle filling. Hence, research on non-spherical particles is increasingly crucial. Chen et al. observed the convection behavior of ellipsoidal particles under vertical vibration, revealing that due to differences in shape and surface characteristics between ellipsoidal and spherical particles, similar convection modes correspond to different vibration parameters. Moreover, under vibration conditions, the alignment direction of ellipsoidal particles tends to become consistent [74]. Yogi et al. investigated the impact of non-spherical particles in binary mixtures on convective vibrational mixing using the discrete element method (DEM). They found that elongated particles mixed best, while cubic particles mixed worst under vibration filling, as illustrated in Figure 9e. Additionally, they calculated the average velocity of coarse particles in binary mixtures, with the overall trend similar to SMI (Ellipsoids > Spheres > Cylinders > Cubes), and higher average velocity of coarse spherical particles indicating higher convection intensity and better mixing outcomes [135]. The mobility of non-spherical particles is usually not as good as that of spherical particles, and the flow process is prone to clogging and uneven filling, which may lead to uneven pressure distribution inside the mold during the compaction process, thus affecting the compaction effect [106]. Currently, it is difficult to accurately predict the flow and stacking behavior of non-spherical particles due to their more complex geometry, which further increases the difficulty of physical and mathematical modeling. Therefore, compared to our understanding of spherical particles, our understanding of the mechanisms of non-spherical particle arrangement and the impact of irregular shapes on convection is not comprehensive, requiring further exploration.

**Figure 10:**
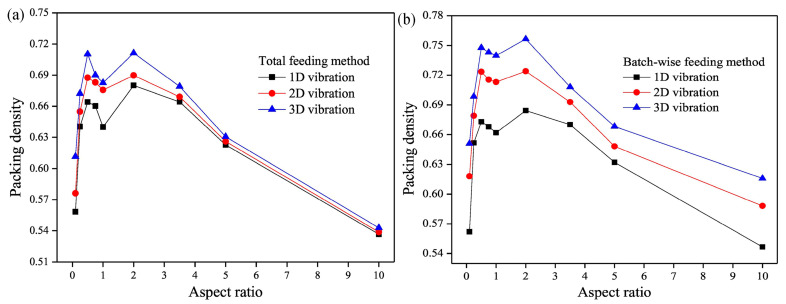
Effects of vibration modes on the stacking density of (**a**) total feeding, and (**b**) batch feeding [86].

To date, most studies on vibration-induced powder particle behavior have focused on vertical vibrations, extensively exploring separation, segregation, and the impact of various parameters on mixtures of vibratory powder particles through experimental and numerical studies. However, research on vibrations in other directions is scarce. Liffman et al. discovered different convection modes from vertical vibrations through horizontal vibration [136]. Under horizontal vibration, Chung et al. also observed the Brazil nut effect within rectangular plates [137]. Further, Li et al. studied the impact of one-dimensional, two-dimensional, and three-dimensional vibration modes on the stacking of ellipsoidal particles, finding that applied vibration significantly increased particle packing density. Vibration effectively eliminated large voids and overcame particle interlocking, forming dense packing. Moreover, compared to one-dimensional and two-dimensional vibrations, three-dimensional vibration achieved higher packing density, as shown in Figure 10. This is mainly because one-dimensional vibration only provides energy for particles in the vertical direction, with horizontal movement restricted. Similarly, two-dimensional vibration only allows particles to move horizontally, not vertically. However, three-dimensional vibration provides energy for particles in both vertical and horizontal directions, thus obtaining a denser packing effect [138]. The study also found that batch feeding achieved higher packing density than full feeding, as full feeding might form more arch structures during filling, resulting in lower packing density. In contrast, batch feeding allows particles more opportunities to move and rearrange, forming more stable positions, especially when vibration is applied.

**Figure 11:**
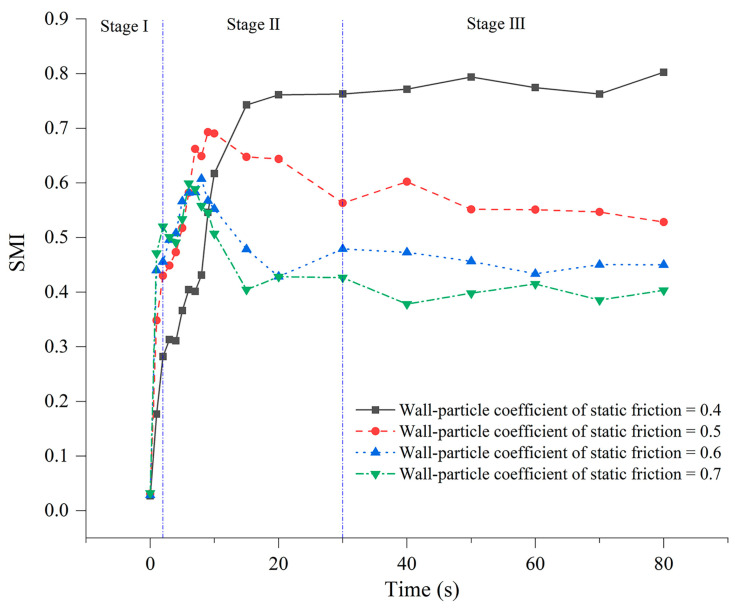
Mixing index versus time curves under different wall friction conditions [134].

Additionally, container size, shape, and wall friction significantly impact the vibrational mixing effect of the powder. Larger container sizes provide more free space for powder particles to move, reducing interparticle interference. During vibration filling, powder particles can move and diffuse more easily, resulting in a more uniform powder distribution. Zhang et al. used the discrete element method (DEM) to simulate the impact of wall friction on the mixing process of binary spherical particles in a vertically vibrated container. The study found that particles near the wall easily moved downward due to wall friction, with particles moving down along the side walls returning to the top from the middle position, forming convection cells. With an increased wall friction coefficient, the macroscopic convection phenomenon became more pronounced. Macroscopic convection caused by wall friction can promote particle flow mixing but may also cause large particles to aggregate at the center of the convection cell, leading to segregation. This phenomenon is particularly evident under different wall friction conditions (as shown in Figure 11). The study showed that, in the first phase of mixing, the variation in the mixing index became more significant with increasing wall friction coefficient, as convection is induced by wall friction [134]. Therefore, the higher the friction coefficient, the more pronounced the convection effect and the clearer the mixing outcome. However, in the third phase of mixing, the mixing index gradually stabilizes, and the degree of segregation increases with the wall friction coefficient. Therefore, to achieve rapid and uniform mixing of particles, it is necessary to consider the wall friction coefficient and other operating conditions comprehensively. An appropriate wall friction coefficient can promote convective mixing while avoiding excessive particle segregation. Hsiau et al. studied the impact of wall friction and container geometry on particle vibration processes, discovering similar convective effects. They also found that the container side wall’s inclination angle significantly affects the direction and flow rate of convection cells. When the side wall’s inclination angle reaches a certain critical value, the direction of the convection cells reverses, meaning particles no longer circulate in the original direction but move and mix in a new direction. As the inclination angle increases, the flow rate of the convection cells gradually decreases [139]. This is because a larger inclination angle increases resistance to particle displacement, restricting free particle flow. Therefore, designing an appropriate side wall inclination angle based on particle characteristics and mixing requirements to promote convective mixing and reduce segregation is one of the key steps to optimizing powder-vibration-filling processes.

### 3.4. Rotary Filling

Rotary filling is extensively adopted within the chemical, food, and pharmaceutical industries, holding an especially vital position in the pharmaceutical sector. Uniform mold filling is crucial, as the quantity of powder in the die determines the weight of the tablet, thereby influencing the drug content and the mechanical-physical properties of the tablet [38,140]. Powder rotary filling is typically an active process, where powder flows into the feeding frame through a hopper and is then pushed into the mold by a paddle feeder, a process we refer to as “an active mold-filling process”.

Accurate mold filling constitutes a complex process, completed under the combined influence of gravity, forced feeding, suction, and rotation [141]. Hence, researchers have extensively studied powder rotary filling to optimize this process. Mendez et al. utilized a combination of a feed frame and a die plate to simulate the powder-filling process, exploring the impact of the operating parameters of the feed frame on the powder-filling process. The feed frame consists of a box with inlets and outlets and several paddles, where powder is poured from the top into the feed frame and then forced out by rotating paddles through the bottom slits, pushing it into the mold. The study found that the weight of powder in the mold increased with the speed of the feed frame and decreased with the speed of the turret and that the powder weight also depended on powder flow characteristics; for example, viscous powders resulted in reduced tablet mass [142], a phenomenon similarly observed by Peeters [143]. For powders with poor flowability, a higher feed frame speed might be necessary for effective filling, while powders with good flowability could achieve uniform filling at lower speeds. However, rotation of the paddles generates shear stress within the powder particles, potentially causing particle abrasion or excessive lubrication, leading to material loss and quality degradation. Ketterhagen et al. evaluated the impact of paddle wheel shapes on mold filling, modeling three different paddle wheel designs. Their study indicated that paddle wheels with larger hubs were preferable, as larger-diameter hubs reduced the amount of powder lingering in the feed frame, lowering the likelihood of severe powder abrasion [144]. Grymonpré et al. designed four different feed frame paddles and evaluated the mold-filling effect, eventually identifying an optimal paddle type [145]. Thus, for different types of powder and process requirements, it is necessary to flexibly adjust paddle designs to ensure optimal filling outcomes. Additionally, the selection of materials for the feed frame and paddles is paramount, as wear-resistant materials can reduce powder particle abrasion and extend equipment lifespan.

In recent years, numerical simulation, particularly the discrete element method (DEM), has played a significant role in analyzing the mechanical behavior of powder during rotary filling and optimizing design. Mateo-Ortiz and Méndez et al. utilized DEM to analyze forces applied to particles during the rotary-filling process, finding that particle translational motion is primarily governed by gravity, tangential forces, and normal forces. The farther the particles from the paddle, the smaller the forces applied to them, because the energy provided by the paddles decreases with distance due to particle collisions, particle friction, and paddle friction [146]. Siegmann et al. reported on different feed frame designs’ flow mixing effects on powder particles using DEM, indicating that optimal geometry shapes and process parameters can be flexibly adjusted based on different powder characteristics [147]. Hildebrandt et al. evaluated the impact of paddle wheel shapes (circular and square) in a three-chamber feed frame on forced filling using DEM. They found that paddle wheel shape significantly affects the filling outcome; cylindrical paddles achieved higher fill quality under low turret-high paddle wheel speeds, while rectangular paddles performed better under high turret-low paddle wheel speeds [148]. An appropriate combination of paddle wheel speeds can yield better filling outcomes, providing a theoretical basis for optimizing paddle wheel design and process parameters.

Despite significant progress in powder-filling technology, many areas warrant further exploration and research, such as developing adjustable feed frames that can flexibly adjust inlet sizes and paddle arrangements based on powder characteristics and process requirements; developing more wear-resistant materials for manufacturing feed frames and paddles; and incorporating machine learning technology to further enhance the efficiency and precision of numerical simulations. In the future, as research in various fields continues to progress, combined with high-precision numerical simulations, more comprehensive and efficient solutions can be provided for optimizing the powder-filling process, expanding the application breadth of powder-filling technology.

Different filling techniques are suitable for different powder characteristics and application demands; these powder-filling technologies each have advantages and limitations. Table 2 summarizes the working principles, applicable powder types, and the advantages and disadvantages of different filling technologies. By choosing the appropriate filling technique, production efficiency, and product quality can be effectively improved, meeting the diversified needs of various industries. With the continuous progress of science and technology, brand-new powder-filling methods are emerging, such as laser powder melting filling [149], ultrasonic-assisted filling [150,151], and so on. These brand-new methods show more possibilities in the field of powder filling, helping to improve production efficiency, product quality, and material utilization. With the continuous development of technology, more innovative filling methods may appear in the future, further promoting industrial upgrading.

## 4. Conclusions and Future Perspectives

The mold-filling process is a critical step in powder pressing and molding, where the uniformity and consistency of powder filling directly impact subsequent processing steps, thus affecting the final quality and performance of powder products. Powder filling into molds is a complex flow process, and accurately assessing powder flowability with appropriate methods has significant implications for product design and process optimization. This article discusses four main mold-filling methods, detailing the flow behavior of powder under different filling mechanisms and analyzing the impact of powder characteristics and processing parameters on filling outcomes. For example, under gravity filling, larger and denser particles achieve a higher mold-filling rate compared to finer particles; for powders with poor flowability, suction filling often yields better results. Therefore, in practical applications, it is essential to flexibly design filling equipment and optimize processing parameters tailored to different powder characteristics and process requirements to better control the filling process.

With the continuous development of new materials and the upgrading of filling equipment, computer modeling still shows enormous potential in assisting the evaluation of process parameters and the design of filling systems. Through computer modeling, the flow and filling effects of powder under different conditions can be simulated and predicted, thus optimizing design schemes, reducing experimental costs, and enhancing production efficiency. Therefore, computer modeling remains an important direction for the future development of powder-filling technology, playing an increasingly significant role in assisting the evaluation and optimization of filling processes. Through ongoing research and innovation, powder-filling technology will continue to advance, providing more efficient and precise solutions across various industries.

## Figures and Tables

**Table 1:** Comparison of different powder flow test methods.

	Test Methods	Measurement Parameter	Evaluation Criterion	Pros and Cons	Applicability for Mold Filling
Traditional Methods	Angle of Repose [68,69,70]	The three-dimensional angle formed by the height and radius of a conical pile of powder	The larger the angle of repose, the worse the flowability	Pros: Broad measurement range, can be used to test powders from free-flowing to very non-flowing Cons: Results are not repeatable, highly influenced by external factors	Does not provide insight into the dynamic flow process, only roughly indicates flow characteristics, not suitable for evaluating powder flow in molds
	Carr Index and Hausner Ratio [71,72,73]	Tapped density, bulk density	The smaller these values, the better the flowability	Pros: Simple, quick, and direct measurement Cons: Highly influenced by external factors	Lacks the dynamic flow process of powder, lacks predictability, not suitable for mold filling
	Orifice Flow Rate [74]	Flow rate through an orifice	The greater the orifice flow rate, the better the flowability	Pros: Powder container shape and orifice are flexible Cons: Difficult to measure non-flowing powders	This test is more for basic flowability assessment of free-flowing powders and is of limited practical help for mold filling
Modern Methods	FT4 Powder Rheometer [75,76,77,78]	Flow function, wall friction, compressibility index	Higher flow function values, smaller wall friction angles, and lower compressibility index indicate better flowability	Pros: Wide testing range, repeatable measurements, high sensitivity Cons: Complex operation, powder samples require pre-treatment	Can simulate the dynamic flowability of powder in actual operations and provide relevant parameters, but its direct predictive capability for mold filling remains limited
	RST Ring Shear Tester [79,80]	Flow function, wall friction force, bulk density, compressibility	Higher flow function values, smaller wall friction angles, and lower compressibility index indicate better flowability	Pros: High accuracy, good repeatability Cons: Not very suitable for viscous powders	Can accurately assess powder flowability and densification, offering some guidance for the mold-filling process, but also has certain limitations
	PFT Brookfield Powder Flow Tester [80,81,82]	Flow function, wall friction, compressibility index	Higher flow function values, smaller wall friction angles, and lower compressibility index indicate better flowability	Pros: Simple operation, reduces operator involvement in the testing process Cons: Shear stress values are fixed and not adjustable	Provides flowability parameters of reference value for mold filling, but the fixed shear stress of the equipment may limit its application across different powder types

**Table 2:** Advantages and disadvantages of different filling techniques and the type of powder to which they are applied.

Filling Process	Principle	Applicable Powders	Advantages	Disadvantages
Gravity filling	Powder naturally flows under the action of gravity	Powders with good flowability, larger particles	Simple operation, low energy consumption, low maintenance cost	Limited filling accuracy, susceptible to environmental influences
Suction filling	Uses vacuum or negative pressure to draw powder into the mold	Fine particles, powders with poor flowability, such as pharmaceutical powders	Precise control of filling volume, strong adaptability	Complex equipment, high maintenance cost, high energy consumption
Vibration filling	Uses vibration to rearrange powder in the mold	Suitable for powders requiring high fill density and uniformity, such as metal and ceramic powders	High fill density, good uniformity, wide applicability	Complex equipment structure, high maintenance cost, noise
Rotary filling	Rotary motion pushes powder into the mold	Suitable for a variety of powders, such as pharmaceutical and food powders	Strong adaptability, uniform filling, high production efficiency	Complex equipment structure, rotary parts prone to wear, high energy consumption

## Data Availability

No new data were created or analyzed in this study.
